# On a New Mode of Treating Severe Dyspepsia and Chronic Inflammation of the Stomach

**Published:** 1859-11

**Authors:** Alexander Fleming

**Affiliations:** Senior Physician to the Queen’s Hospital, Birmingham


*On a New Mode of treating severe Dyspepsia and Chronic Inflammation of the Stomach.* By Dr. ALEXANDER FLEMING, Senior Physician to the Queen's Hospital, Birmingham.

In the medicinal treatment of affections of the stomach, I have long been convinced of the great importance of acting directly on the gastric mucous membrane. That, in fact, local treatment is here nearly as valuable as it is in other affections of mucous surfaces, as the eye, pharynx, vagina, and urethra. Hygienic rules, and the management of the food, are, for obvious reasons, very important in affections of the stomach, and will often cure mild cases without the help of medicine; but I am satisfied that, in the more severe and obstinate forms of chronic gastritis, the local medicinal treatment of the diseased mucous membrane has been unduly neglected, and that it contributes very powerfully to promote the cure.

Of the several medicines which I have employed with a view to their *local* action on the stomach, my experience gives the first place to nitrate of silver; and the observations I have now to make apply to this remedy. It is often given in pill. If this be made with bread-crumb, the chloride of sodium in the bread converts the nitrate into the insoluble and comparatively inert chloride of silver. If made with gum or starch, the pill, on reaching the stomach, causes quickly the secretion of gastric juice, the chloride of sodium and muriatic acid of which again render the nitrate inert. It can have very little *local* action in the form of a pill. I have for many years, therefore, given the crystallized nitrate dissolved in distilled water, in the proportion of from ½ grain to four grains to the half-ounce. The dose is taken at bed-time on an empty stomach, and is repeated every night, every second, third, or fourth night, according to the severity of the disease. The stomach should be strictly empty—the patient recumbent—and he should be made to roll about immediately after taking the medicine. It is thus—before it suffers decomposition—brought into contact pretty freely with the mucous membrane, and gives, at the time, and subsequently, evidence of its local action. In many cases, this mode of using the remedy suffices, in conjunction with other means, to effect a cure.

But this method of exhibiting the medicine is not equal to the cure of some of the severer forms of dyspepsia and chronic gastritis; and in these, I have, for the last four years, endeavored to act more generally and efficiently on the mucous surface by injecting the solution into the stomach. I employ a strong brass syringe and flexible tube—one-eighth of an inch in bore—the gastric end of which has a number of holes so directed that the fluid is thrown in a circular shower outwards and upwards on the walls of the stomach. The injection is made by dissolving from one to four grains of the nitrate in three ounces of distilled water. The operation is for the most part managed easily. Sometimes it causes nausea and retching—oftener not. It excites at first an enduring and grateful sense of coolness in the stomach; and subsequently there are felt pricking and sharp painful sensations, but of a different nature from the pains of the disease. Sometimes one injection is enough; but I have more frequently had to repeat it two, three, or more times.

During the employment of the injections, the patient takes three times a-day and before food a little morphia or chloric ether, or Indian hemp, in plain or cinnamon water. He is confined to small and frequent meals of milk; and as he gets better this is thickened with arrow root or tapioca, and he is very gradually introduced to a nourishing and easily digestible diet. Counter-irritation to the epigastrium, of bismuth, oxide of silver, gentle tonics, &c., are employed when indicated.

Of the thorough efficiency of this mode of acting on the mucous surface of the stomach, and of its power in promoting the cure, my experience, so far as it goes, is very decided. Although it is now four years since I first tried injection, I have not used it in more than ten cases. I have always, in the first instance, employed the simpler method already described, and resorted to injection only as a last resource; but its greater efficiency would, I feel certain, justify its employment in many of the less severe cases, and give more thorough and speedy cures. It is not my purpose at present, to consider the intimate nature of the mode of cure, or the manner in which the nitrate of silver substitutes healthy for diseased action in the inflamed gastric membrane. I must reserve that interesting question, and the detailed narrative of cases for another opportunity.

Mr. Young, cutler, of Edinburgh, made the instrument I now use for injecting the stomach, and it answers extremely well.—*Med. Times and Gazette, Jan.* 29, 1859, *p.* 108.

